# Vascular Parkinsonism: Still Looking for a Diagnosis

**DOI:** 10.3389/fneur.2018.00411

**Published:** 2018-06-15

**Authors:** Giovanni Mostile, Alessandra Nicoletti, Mario Zappia

**Affiliations:** Section of Neurosciences, Department “G.F. Ingrassia”, University of Catania, Catania, Italy

**Keywords:** vascular parkinsonism, diagnostic criteria, Magnetic Resonance Imaging, SPECT, CSF monitoring, vascular-PSP, Normal Pressure Hydrocephalus

## Introduction

Vascular Parkinsonism (VP) is a clinical entity generally defined as a parkinsonian disorder temporally-related or associated with ischemic cerebrovascular disease.

Available proposed diagnostic criteria for VP are based on pathological data by Zijlmans et al. (1). In particular, the diagnosis of “*vascular parkinsonism of insidious onset*” is supported by the presence of: (a) parkinsonism; (b) relevant cerebrovascular disease by brain imaging; (c) insidious onset with extensive subcortical white matter lesions, bilateral symptoms at onset, and the presence of early shuffling gait or early cognitive dysfunction. This common form should be distinguished from the “*acute or delayed progressive onset form*” with a closed relationship between clinical asymmetric signs, sites of lesion and stroke onset (1).

A recent update of the diagnostic approach for a subtype definition of VP proposed also a third subgroup for classification of VP, defined as “*mixed neurodegenerative parkinsonism and cerebrovascular disease*,” to be used when a diagnostic overlap with neurodegenerative disease is suspected (2).

The clinical heterogeneity which characterize VP patients lead to a significant diagnostic overlap with idiopathic Parkinson's Disease (PD) and other atypical or secondary parkinsonian disorders, including Progressive Supranuclear Palsy (PSP) and idiopathic Normal Pressure Hydrocephalus (iNPH). Until now, there are however few instrumental supporting information by neuroradiological or neurophysiological biomarkers which may help physicians in ameliorate diagnostic accuracy when VP is suspected. In this paper, we will discuss possible approaches for the differential diagnosis between VP, PSP, and iNPH, providing some clinical examples.

## VP vs. PD

### Clinical vignette (case #1)

A 75-year-old woman with a 4-years history of progressive gait difficulties and generalized slowness. Neurological examination revealed the presence of a parkinsonism characterized by wide-based cautious gait and generalized bradykinesia and rigidity, more prominent on the left arm. Brain Magnetic Resonance Imaging (MRI) scans showed multi-infarctual leukoencephalopathy involving the basal ganglia.

Differential diagnosis with PD represent the first step when evaluating patients with a possible diagnosis of VP. Despite the clinical overlap, case-series descriptions have highlighted the presence of a pronounced lower-body involvement with postural instability and falls, a more frequent symmetric and akinetic-rigid presentation, additional features including cortico-spinal and pseudobulbar signs, urinary incontinence and cognitive decline in VP as compared to PD patients (3).

Patents with VP may have a response to L-dopa treatment in almost 30% of cases (3–6), sometimes reporting long-term motor fluctuations possibly due to cerebral ischemic lesions closely related to nigrostriatal pathway. This may lead to additional issues in differential diagnosis with idiopathic PD, considering that the presence of a sustained L-dopa response as well as the detection of L-dopa induced motor fluctuations and dyskinesia represent hallmarks for the diagnosis of idiopathic PD (7).

Nevertheless, few study systematically investigated dopaminergic responsiveness in VP, both acutely and chronically. It should be noted that, beside to the motor improvement induced by L-dopa, other aspects could be of interest. For instance, possible predictors of poor tolerability should be searched, in order to assess possible differences with data reported for PD (6, 8). Moreover, patient tolerability to the L-dopa short-term test may give additional information for distinguish VP from idiopathic PD, since side effects have been more frequently recorded with almost a double occurrence among patients with atypical parkinsonian disorder as compared with PD (9). Information on pharmacological response in VP is then crucial for ameliorating diagnostic accuracy.

Functional neuroimaging is required to evaluate the integrity of the nigrostriatal system in the clinical context of a parkinsonism. Single photon emission computed tomography (SPECT) study in VP may reveal abnormal striatal dopamine transporter binding in almost 70% of patients (5), with a common symmetrical basal ganglia uptake reduction (10). A quantitative approach using single photon emission computed tomography (SPECT) with [123I]FP-CIT based on ligand uptake in specific regions of interest has been used to compute a Striatal Asymmetry Index (SAI), which demonstrated to differentiate with high specificity VP from PD (11). Moreover, in patients with PD SAI values were correlated with magnitude of the acute motor response to L-dopa (12).

Other clinical and laboratory findings may be also useful if integrated to the cardinal clinical features to distinguish VP from PD. Hyposmia, rapid eye movement sleep behavior disorder, slow colonic transit time as well as cardiac reduction in metaiodobenzylguanidine cardiac uptake using SPECT imaging have been in fact associated with PD. They were instead not systematically reported in patients presumed to have VP (13).

### Case #1 diagnosis

Semiquantitative analysis of DaT-SCAN SPECT imaging showed a bilateral reduction in striatal tracer uptake, prominent on the right striatum, in accordance with clinical lateralization. A SAI of 15.1 was estimated, as observed in PD (cut-off vs. VP: 14.08) (11). L-dopa treatment was started, with evidence of a good chronic response at the follow-up visits. Despite the documented extensive cerebrovascular disease in the clinical context of a parkinsonism may suggest a diagnosis of VP with “insidious onset” (1), in this subject functional imaging data cannot allow to exclude a neurodegenerative process compatible with PD.

## VP vs. PSP

### Clinical vignette (case #2)

A 67-year-old man with a 2-years history of progressive gait difficulties and generalized slowness. Neurological examination at the first visit revealed the presence of parkinsonism characterized by postural instability with falls, wide-based cautious gait and generalized bradykinesia, long-latency saccades with restricted vertical gaze range of motion and mild dysarthria. Brain MRI scans showed multi-infarctual leukoencephalopathy involving the basal ganglia. L-dopa therapy was started, but treatment was discontinued 6 months later for lack of benefit.

As already stated, the diagnostic characterization of patients with VP is complicated also by a diagnostic overlap with other atypical parkinsonian disorders. Specifically, VP patients may share clinical cardinal features with PSP patients with parkinsonian features (PSP-P), including marked postural instability and falls (3).

In VP patients, unilateral mesencephalic reticular formation or bilateral thalamic lesions have been identified as potential causes of vertical nuclear ophthalmoplegia mimicking the classic vertical supranuclear ophthalmoplegia of PSP (14). Unfortunately, there are still no clinically defined diagnostic characteristics which can discriminate PSP-P from VP patients (15). Indeed, within the first 2 years of disease, fewer than one third pathologically proven PSP patients could exhibit supranuclear gaze palsy and only approximately half of them could have falls (16). Even more, in some PSP cases, supranuclear gaze palsy may be only observed in the later stages of the disease (17).

There are still also controversies on pathological basis differentiating the two conditions, which are based on studies documenting the presence of cerebrovascular lesions in PSP (14, 18). Results may support the notion that unspecified vascular lesions detected by MRI scan could not exclude that patients may meet pathological diagnostic criteria for idiopathic PSP instead of VP if sharing common clinical features.

Differential diagnosis may result also difficult in patients presenting freezing of gait as major clinical feature at the disease onset in the context of a cerebrovascular disease, since in this case VP should be distinguished from the PSP-form presenting with pure akinesia and freezing of gait (19).

Morphometric measures obtained by neuroimaging study have shown to be helpful in the differential diagnosis. Morphometry analysis of selected brain structures using conventional MRI, specifically midbrain and superior cerebellar peduncle, pons and middle cerebellar peduncle, when combined to compute a Magnetic Resonance Parkinsonism Index (MRPI), may be a reliable tool to differentiate PSP from PD and other atypical parkinsonian disorders (20–22). MRPI has been shown to be also helpful when applied in the differential diagnosis between VP and PSP, discriminating with high accuracy the two conditions when a cut-off value of 13 was applied (23).

### Case #2 diagnosis

Based on brain MRI scans, computed MRPI was 6.8, suggesting a diagnosis of VP with “insidious onset” instead of PSP (1, 23). Comparing to the first observation, a 6 years follow-up visit revealed a slow progressive clinical course with a general stability of the neuroradiological aspects. In this case, the relative mild disease course together with MRI findings may suggest VP as reasonable clinical diagnosis.

## VP vs. iNPH

### Clinical vignette (case #3)

A 65-year-old man with progressive gait difficulties causing frequent falls. Some memory complains were also referred. Neurological examination revealed the presence of a symmetrical akinetic-rigid parkinsonism with postural instability and a “magnetic gait” with start hesitation. Brain MRI scans showed leukoaraiosis and ventriculomegaly with a moderate degree of global cortical atrophy.

**Figure 1:**
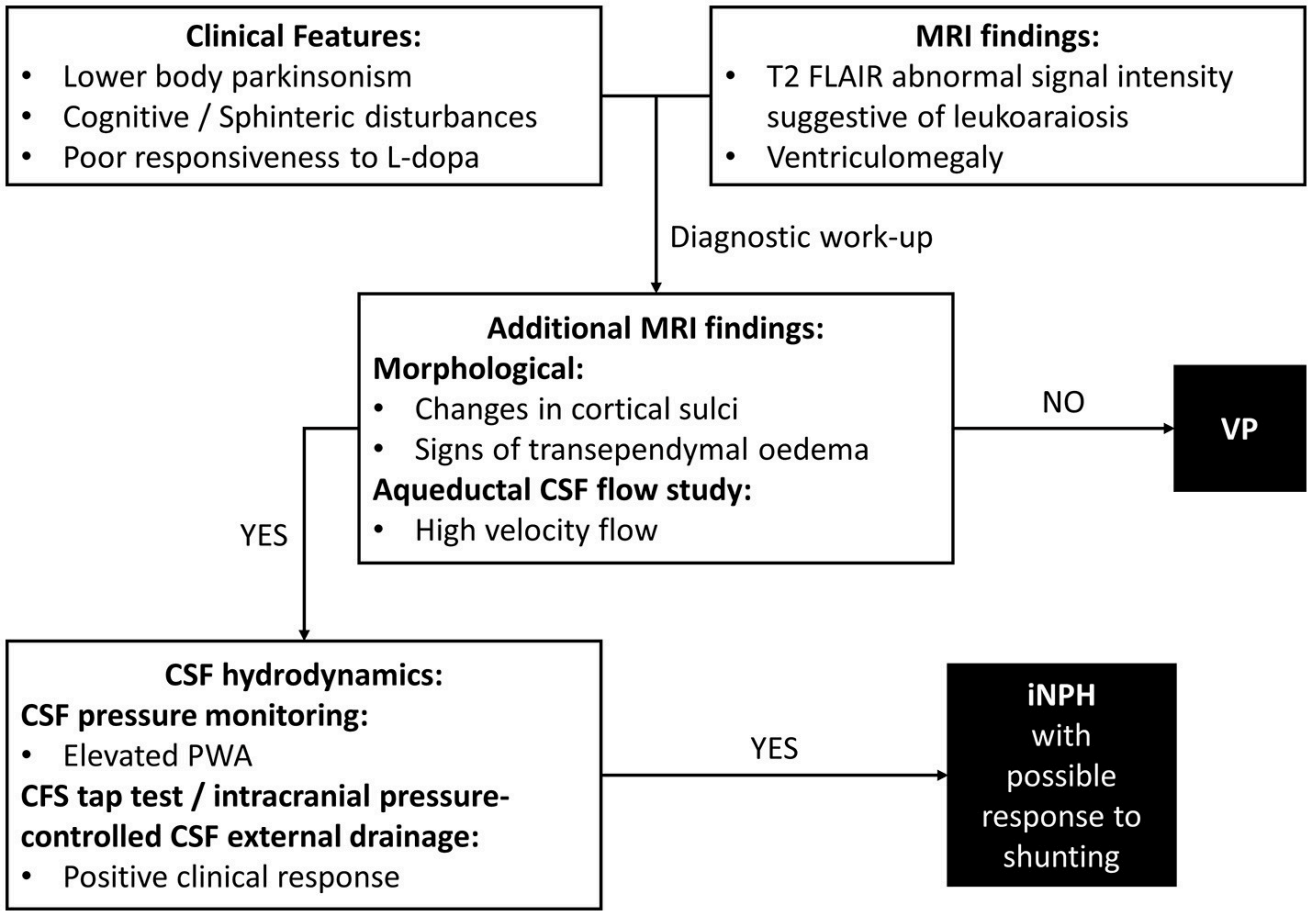
Differential diagnosis of VP. Diagnostic steps approaching a case of suspected iNPH. VP, Vascular Parkinsonism; iNPH, Normal Pressure Hydrocephalus; MRI, Magnetic Resonance Imaging; CSF, Cerebrospinal Fluid; PWA, Pulse Wave Amplitude.

In VP patients, beside to ischemic cerebrovascular lesions, neuroimaging may highlight ventricular enlargement (3). This radiological feature is distinctive of iNPH. iNPH is clinically characterized by gait disturbance, cognitive impairment and urinary incontinence, but parkinsonian features could be also common, making differential diagnosis between VP and iNPH particularly challenging (24). Diagnosis of iNPH is focused on radiological evidence of enlarged cerebral ventricles with normal Cerebrospinal Fluid (CSF) pressure. In clinical practice, the diagnostic approach needs to combine clinical, neuroradiological and CSF hydrodynamic data (Figure 1). To date, there are few distinctive tools which may be useful in identifying patients with iNPH. Improvement after ventricular shunting in iNPH remains still variable and thus not diriment in the differential diagnosis with VP, even though there are potential clinical and instrumental predictors of a successful outcome of shunting which include age, response to external lumbar drainage or tap test and CSF pulsatility monitoring (25).

Diagnostic tools to differentiate VP from other secondary parkinsonian disorders as iNPH need to be then investigated. It has been reported that CSF hydrodynamic analysis assessed through Pulse Wave Amplitude (PWA) evaluation could predict the clinical response to surgical treatment in iNPH (26). PWA represents the intracranial pressure pulsation closely related to systo-diastolic components of arterial pressure and it is considered helpful to estimate intracranial compliance (27). CSF pressure components in patients with clinical aspects compatible with VP and brain ventricular enlargement has been investigated, showing elevated PWA values during a CSF pressure monitoring as observed in iNPH patients responsive to shunt implantation (27). Moreover, it has been reported that some patients with clinical and radiological features of VP could improve after a 3-day external lumbar drainage procedure (28). Thus, it could be hypothesized that some patients with apparent VP and brain ventricular enlargement could be affected by iNPH, eventually improving after ventricular shunting. On these grounds, tools to discriminate between VP and iNPH should be welcomed.

### Case #3 diagnosis

Additional morphological data on brain MRI included crowding of the gyri at the vertex, enlargement of Sylvian fissures and signs of transependymal oedema (29). MRI aqueductal CSF flow study indicated high-velocity aqueductal flow (25). CSF pressure monitoring data showed a mean PWA value of 68.5 mmH_2_O (normal values ≤ 54.8 mmH_2_O), as observed in iNPH (27). A clinical response to intracranial pressure-controlled CSF external drainage was documented (25). Taken together, clinical-instrumental information made the diagnostic suspect of iNPH possibly responsive to shunting clinically reasonable (Figure 1). A ventriculoperitoneal shunt procedure was then perfomed with documented clinical benefits at the follow-up visits.

## Final considerations

Considering all criticisms in the diagnostic overlapping as already discussed, it has been proposed to use, instead of the term “VP,” clinical descriptors including “*pseudovascular parkinsonism*” (neurodegenerative parkinsonism with non-specific neuroimaging signal abnormalities), “*vascular pseudoparkinsonism*” (e.g., akinetic mutism due to bilateral mesial frontal strokes or apathetic depression from bilateral striatal lacunar strokes), or “*pseudovascular pseudoparkinsonism*” (e.g., higher-level gait disorders, including iNPH) (30). Nevertheless, clinical descriptors as those above mentioned do not allow differentiation among different conditions, such as VP, PD, PSP, and iNPH.

Therefore, there is a need for studies looking at biological biomarkers, in order to define an integrated clinical diagnosis by instrumental supports. An updated diagnostic approach proposing three different diagnostic subgroups (“*acute/subacute VP*,” “*insidious VP*,” and “*mixed neurodegenerative parkinsonism and cerebrovascular disease*”) has been formulated by an expert panel and integrated by qualitative supporting information by conventional MRI and SPECT studies, with the proposal of a prospective validation of the proposed diagnostic approach (2). However, a combined clinical-instrumental approach using quantitative indexes which have been already tested for the differential diagnosis of VP, including SAI by [123I]FP-CIT SPECT, MRPI by morphometric MRI, and PWA by CSF pressure monitoring, could be proposed for the definition of new combined and integrated diagnostic criteria to be validated.

## Author contributions

GM, AN, and MZ wrote the first draft and revised it critically.

### Conflict of interest statement

The authors declare that the research was conducted in the absence of any commercial or financial relationships that could be construed as a potential conflict of interest. The reviewer CC declared a past co-authorship with several of the authors AN and MZ to the handling Editor.
